# Geoarchaeological evidence from Angkor, Cambodia, reveals a gradual decline rather than a catastrophic 15th-century collapse

**DOI:** 10.1073/pnas.1821460116

**Published:** 2019-02-25

**Authors:** Dan Penny, Tegan Hall, Damian Evans, Martin Polkinghorne

**Affiliations:** ^a^School of Geosciences, The University of Sydney, Sydney, NSW 2006, Australia;; ^b^École Française d’Extrême-Orient, 75116 Paris, France;; ^c^Archaeology, College of Humanities, Arts and Social Sciences, Flinders University, Adelaide, SA 5001, Australia

**Keywords:** Angkor, collapse, Cambodia, archaeology

## Abstract

Alternative models exist for the movement of large urban populations following the 15th-century CE abandonment of Angkor, Cambodia. One model emphasizes an urban diaspora following the implosion of state control in the capital related, in part, to hydroclimatic variability. An alternative model suggests a more complex picture and a gradual rather than catastrophic demographic movement. No decisive empirical data exist to distinguish between these two competing models. Here we show that the intensity of land use within the economic and administrative core of the city began to decline more than one century before the Ayutthayan invasion that conventionally marks the end of the Angkor Period. Using paleobotanical and stratigraphic data derived from radiometrically dated sediment cores extracted from the 12th-century walled city of Angkor Thom, we show that indicia for burning, forest disturbance, and soil erosion all decline as early as the first decades of the 14th century CE, and that the moat of Angkor Thom was no longer being maintained by the end of the 14th century. These data indicate a protracted decline in occupation within the economic and administrative core of the city, rather than an abrupt demographic collapse, suggesting the focus of power began to shift to urban centers outside of the capital during the 14th century.

## Significance

Contrasting models exist to explain the movement of urban populations following the 15th-century demise of Angkor. Here we present geoarchaeological data from the urban core of Angkor that indicate a protracted decline in land use intensity during the 14th century rather than an abrupt demographic collapse. These results argue against traditional explanations for the demise of Angkor, which emphasize the role of interventionist foreign powers in forcing collapse, and imply a more complex and protracted transformation.

**Fig. 1.**
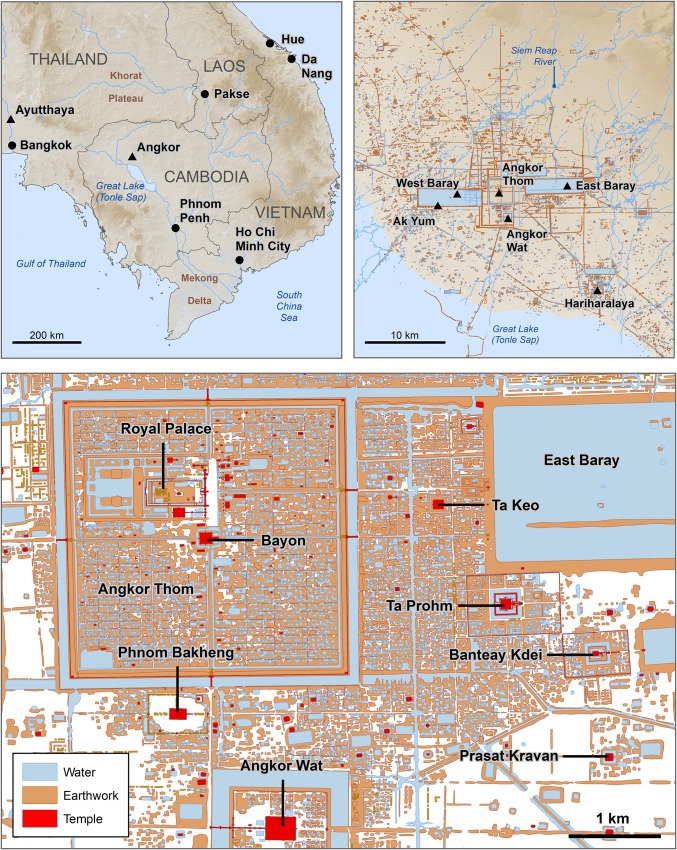
Location map of Angkor, and the location of sites mentioned in the text.

The demise of Angkor (Fig. 1) remains poorly understood. The paucity of epigraphic evidence and monumental construction after the 13th/14th century CE means that many accounts of this period are vague and speculative. One narrative identifies the sack of Angkor by Thai forces from Ayutthaya in 1431 CE as the *coup de grâce*—“definitive, complete, and irremediable” (ref. 1, p. 258)—preceded by a protracted decline in state power and influence from the 14th century CE. Although Ayutthayan forces are believed to have occupied Angkor for a period between 12 y and 15 y, historical sources are both equivocal and contradictory regarding these events (2). Cambodian narratives on collapse transformed over many hundreds of years and are largely institutional and community constructions that emerged between the 16th and 19th centuries (3, 4). Moreover, there is little reflection on the mechanics of demographic change despite known continuity of residential occupation and substantial building works into the 16th century CE (5–9).

Numerous hypotheses have been proposed to account for Angkor’s decline. Groslier (10) suggested the capital collapsed because of the deterioration of the irrigation system and systemic environmental degradation associated with extensive forest clearance (11, 12). Others have emphasized the supposed economic and social distress caused by the Jayavarman VII’s “building orgy” (ref. 1, p. 258), and the torpor associated with the rise of Theravāda Buddhism (ref. 13, p. 497; ref. 14, p. 224). Still others claim the growing lure of maritime trade in the Mekong Delta may have eroded the power base of Angkor (2), or that climate change (15–18) or epidemic disease (19, 20) may have contributed. All of these explanations are problematic, however, and none have proven decisive (21).

Lucero et al. (22) argue that the Khmer elite, like those in other tropical low-density cities had, by the 16th century, relocated to small and burgeoning *entrepôts* on the fringes of the ancient agrarian kingdom. In their view, “the low density dispersed urban landscape faded away and the urban world re-created itself in a more compact form in new locations and regions along the peripheries. This movement across the landscape we term *urban diaspora*, when people abandon not only the urban centers but much of the metropolitan heartland and move to peripheral areas where different kinds of networks and economic and political foci emerge” (ref. 22, p. 1139–1140). Evans (23) takes a contrary view, arguing that the demise of Angkor as the locus of power “may not, in fact, have involved the physical ‘movement’ of anything much at all, let alone radical demographic shifts” (ref. 23, p. 9). Rather, he suggests continuity and fluidity of power between royal houses, and a slow rather than catastrophic decline in urban population.

Whether the supposed “urban diaspora” associated with the demise of Angkor presaged or followed the administrative and commercial demise of the city is a matter of speculation, as is the speed with which that process occurred. This is a potentially decisive issue in revealing the process by which large low-density cities like Angkor ultimately fail, and one that can be addressed empirically. This paper will provide a radiometrically dated record of land use from an area of central Angkor that was a focal point for administrative and commercial activities from the early 10th century onward. Using these data, there are two competing models we seek to evaluate: first, a relatively abrupt (decadal scale) exodus of people from the core area precipitated by discrete periods of hydroclimatic change and social upheaval in the 14th and 15th centuries, caused by the dependence of the elite on the inflow of surplus agricultural yield from the countryside, and, alternatively, a more gradual (centennial scale) decline in the elite, non-rice-producing population that may be asynchronous with periods of hydroclimatic instability, and which may point to the primacy of exogenous forces and a slower, more complex transformation in the Cambodian state.

Here we interrogate land use-sensitive proxies measured from drill cores of sediment accumulated within the encircling moat of the 12th-century citadel of Angkor Thom. Variations in land use over time, inferred from these proxies, reflect patterns of occupation and abandonment and can be absolutely dated. Centered on the area surrounding the southern gate to Angkor Thom, this area has been a site of occupation for millennia and sits at the center of Angkor’s administrative and political operations (see *SI Appendix* for more detail).

## Result

**Fig. 2.**
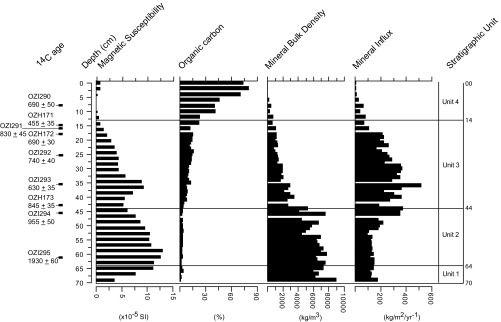
Stratigraphic and sedimentological data for core AT/01/04/B, plotted against depth. Stratigraphic units are identified based on changes in color and texture.

Summary stratigraphic data are plotted against depth in Fig. 2 and are described in *SI Appendix*. Volume magnetic susceptibility, expressed as a dimensionless ratio value in the international system of units (SI), is low and variable in all cores (ranging from an average of 2.97 ± 3.26 × 10^−6^ SI for core AT/01/06/B, to 5.39 ± 4.16 × 10^−6^ SI for core AT/01/06/A), reflecting the diamagnetic properties of the primarily siliciclastic or biogenic sediment. Sequence slotting demonstrated strong correlation between the four cores based on their magnetic susceptibility profiles (Delta is 0.15 between AT/01/06/B and AT/01/06/A, and 0.25 between AT/01/06/B and AT/01/04/B; Rp = 0.999, Rs = 0.974). This indicates that all cores are representative of the stratigraphy at the core site, allowing an analytical focus on one core only. Core AT/01/04/B (70 cm in length) was chosen for this purpose.

Results of radiocarbon analyses are presented in *SI Appendix*, Table S1. The Hohenheim wood standard (OZI296) returned an age of 2,210 ± 40 ^14^C y B.P., closely coherent with its radiocarbon age 2,215 ± 5 ^14^C y B.P., and its weighted average consensus age of 2,232 ± 5 y B.P. Accordingly, contamination of the samples during the extensive pretreatment procedure can be disregarded.

OZI295, taken toward the base of unit 1 (61 cm to 62 cm depth), returned a calibrated age in the first century CE, suggesting that this mottled sandy clay is the substrate into which the moat of Angkor Tom would be cut, or is reworked from same. OZI294 and OZH173 bracket the abrupt boundary between units 2 and 3 (44 cm depth) and return ages of the late 11th and late 12th centuries, respectively, in good agreement with the epigraphic age for the enclosure (24). The change in color, magnetic susceptibility, organic carbon, mineral bulk density, and mineral influx at this boundary (Fig. 2) reflects the establishment of reducing conditions and better preservation and/or greater input of biomass to the sediment. This pattern is consistent with the excavation of temple moats and groundwater fed reservoirs at Angkor (25). OZH173, taken from the base of unit 3 and thus representing the earliest organic sedimentation with the newly excavated moat, was deposited between 1051 CE and 1264 CE (weighted mean probability of 1197 CE; *SI Appendix*, Table S1). This coincides closely with the coronation of Jayavarman VII (reign 1182/3 CE to 1220 CE) suggesting, as Cœdès (ref. 24, p. 89) and Jacques (ref. 26, p. 45) argue, that the building of the Angkor Thom enclosure wall, and the excavation of its moat, was undertaken early in Jayavarman’s reign. This is, however, earlier than the date proposed by Gaucher (27) for Jayavarman’s final remodeling of the moat, and closer in age to the deepening of the moat in Gaucher’s (28) “phase 2” period between the 11th century and the end of 12th century.

There are a number of age inversions in the middle of the sequence, and the probability distributions of three of these ages (OZI293, OZI291, and OZH171) are effectively ignored (or “bypassed”; ref. 29) in the calculation of the chronological model (*SI Appendix*, Fig. S1).

**Fig. 3.**
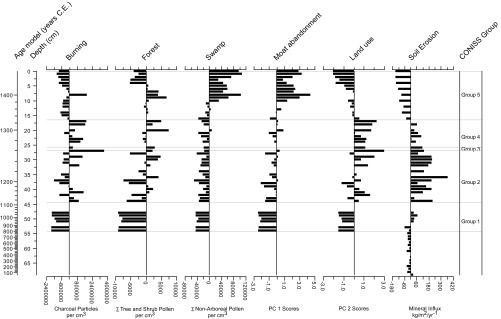
Summary stratigraphic, microbotanical, and numerical results, plotted around the long-term mean value for each variable, against modeled age and depth.

Forty-eight pollen and spore samples were analyzed, producing a dataset with 194 variables (taxa). Seventy-seven palynomorph types were unidentified, representing, on average, 1.55% of each sample. The number of individual palynomorphs ranged from a minimum of 19 (53 cm to 54 cm depth) to a maximum of 2,285 (0 cm to 1 cm depth), with a mean of 310 individuals per sample. A total of 14,997 individuals were recorded. A description of the palynological and microcharcoal data is given in *SI Appendix*. Taxa are expressed as absolute abundances against depth in *SI Appendix*, Fig. S2, and as variance around the long-term mean, grouped into broad habitat classes, in Fig. 3. Stratigraphically constrained classification identified five sample groups (0 to 16, 17 to 26, 27, 28 to 44, and 48 to 54 cm depth; *SI Appendix*, Fig. S3).

Principal Component Analysis (PCA) indicates that the first two principal components account for 53% and 30%, respectively, of the variance in the data set (*SI Appendix*, Fig. S4*A*). Scree plot, in comparison with a broken stick model (*SI Appendix*, Fig. S4*B*), suggests that only the first two components are significant. Variable loading scores indicate that charcoal concentrations (0.72) and arboreal (trees and shrubs) pollen concentrations (0.58) most strongly influence the distribution of sample scores along the second component axis, while pollen from local sources, reflecting the occupation of the moat by swamp vegetation, most strongly influences the distribution of sample scores along the first component axis (herbs, aquatics, and ferns have loading scores of 0.51, 0.57, and 0.52, respectively).

## Disturbance and Land Use

The findings described in *Result* indicate that the moat of Angkor Thom was excavated between the end of the 11th century and the end of the 12th, and indicate a modeled age of 1125 CE. From that time, the surrounding dryland vegetation was characterized by a changing admixture of dry forest and cultivated plants, with no clear dominant taxon. The herbaceous and aquatic assemblage is dominated by grasses and sedges and a relatively limited representation of typical herbaceous swamp (Chenopodiaceae/Amaranthaceae group, which is here dominated by *Alternanthera* pollen, as well as *Ludwigia* and *Persicaria*) and obligate aquatic taxa (*Ceratophyllum* leaf spines). Regular, probably low-intensity burning is clearly important, with sedimentary charcoal being highly variable around the long-term mean from the early 12th to the late 14th centuries. It is not surprising, then, that indicators of disturbance (possibly Urticaceae/Moraceae but particularly *Macaranga*) are important in the dryland pollen fallout during this period, and particularly so from the 1270s CE (25 cm depth).

Sedimentation within the moat was dominated by siliciclastic materials from its excavation until the last decades of the 14th century, when organic sediment derived from encroaching swamp vegetation began to dominate. Mineral influx values remained high and variable until the mid/late 13th century. The rate at which mineral sediment accumulated in the moat can be taken as a proxy for the rate of supply, there being no authigenic minerals present in the sediment. The pathways for supply of mineral sediment to the moat include erosion of the surrounding banks (although the banks and causeways are encased in laterite and sandstone steps), deposition of fines from within the city itself through drainage into the southwest corner of the encircling wall, and windblown sediment, primarily from the proximal areas kept free of vegetation due to continual use (such as roadways and market areas, for example), but also small amounts of dust fallout from regional sources. Increased supply of mineral material to depositional sinks around Angkor Thom reflects disturbance and use, either through construction, vegetation removal (directly or through burning), or agriculture.

The relationship between the rate of supply (controlled by land use) and the rate of mineral accumulation in the moat is potentially compromised by the development of local aquatic vegetation which can “isolate” the sediment from mineral input by trapping mineral sediment at the moat margins or on floating vegetation mats. This potentially confounding influence is most likely in the upper 14 cm of the core (stratigraphic unit 5, a modeled age of 1340 CE), and particularly in the upper 6 cm of the core (*ca*. 1420 CE) where the presence of fibrous peat and an average organic content above 68% by weight (Fig. 2) indicates unequivocally the presence of a floating mat of swamp vegetation at the core site. Mineral influx and organic carbon values are, however, not significantly correlated down core (*r*^2^ = 0.21), nor are inorganic and organic influx values (*r*^2^ = 0.146). If the development of floating vegetation were trapping mineral sediment, then one would expect a strong negative relationship between these variables. This suggests that change in mineral flux to the core site is indeed controlled by the rate of supply and is not influenced by mineral sediment being trapped by local swamp vegetation.

Decomposition of the data by PCA reveals that the second principal component scores (PC2) most strongly reflect variation in burning and dryland vegetation in central Angkor. The plot of these scores against time can, therefore, be used as a proxy for land use intensity from the 12th century (Fig. 3). PC2 scores decline sharply and consistently from the early 14th century (1316 CE or 17 cm depth). During that time period, mineral influx (reflecting soil disturbance) and PC2 scores (reflecting, primarily, fire frequency and vegetation disturbance) are very strongly correlated (*r*^2^ = 0.88; *n* = 9), but the fact that the correlation between these two variables is much weaker for all samples (*r*^2^ = 0.27; *n* = 38) suggests a strong and consistent response to changes in land use in central Angkor from the early 14th century. Below 17 cm depth (a modeled age of 1316 CE), the PC1 and PC2 scores are strongly correlated (*R*^2^ = 0.87). Above 17 cm depth, the two axes scores become weakly anticorrelated (*R*^2^ = 0.13), reflecting the increasing importance of swamp vegetation in influencing PC1, which appears to be quite independent of changes in land use intensity suggested by PC2. In fact, it is not until 11 cm depth (*ca*. 1374 CE) that significant changes in PC1 scores are apparent. This, in turn, suggests that occupation of the moat of Angkor Thom by floating swamp vegetation occurred more than half a century after the first indications for a decline in land use intensity in the dryland pollen and charcoal data. Regardless, it is clear that proxy indicators for fire and dryland vegetation changing in concert with proxies for soil disturbance and occupation from the early 14th century indicate a clear and consistent decrease from that time.

## Decline Versus Collapse

Here we present sedimentological and palynological data from central Angkor that reflect changes in land use intensity over time. Prima facie, these data imply that land use intensity in the administrative and commercial core of Angkor declined progressively from the first decades of the 14th century, rather than catastrophically as a result of a demographic “collapse” associated with the Ayutthayan occupation of Angkor one century later. The last known inscription associated with the consecration of a monument of Angkor is dated to 1295 CE, at the Maṅgalārtha temple within Angkor Thom, and the data presented here suggest that land use began to attenuate around the south gate of Angkor Thom only two decades later. By the end of the 14th century, the southern moat of Angkor Thom was overgrown with vegetation, and management, by implication, had ceased. The hypothesized fragmentation of Angkor’s water management network (11) and the climatic variability that likely triggered that fragmentation (17) occurred in this changing administrative and political context.

Evidence for a gradual decline in the vibrancy of Angkor’s administrative core is consistent with an emerging consensus that multiple factors coalesced to encourage the Cambodian elite to relocate closer to the Mekong and Tonle Sap River (30). Not least among these factors was the burgeoning territorial ambitions of neighboring states, better access to profitable maritime trade networks closer to the South China Sea, and an increasingly unwieldy and restrictive urban fabric at Angkor. Our data support the interpretation offered by Evans (23) that the demise of Angkor was characterized by a “gradual demographic decline” (ref. 23, p. 172) rather than the sudden mass movement of large urban populations envisaged by Briggs (1), or the “disappearance” of the residential population implied by Lucero et al. (22). An additional complexity here is that a decline in land use in the urban core presupposes an analogous decline in land use in the much larger agricultural landscape. However, Groslier (31) argued against this, suggesting that pre-Angkor period patterns of agricultural land use reasserted themselves after the demise of Angkor’s administration, suggesting a deep resilience in the low-density, productive landscape.

Comparative data from secondary cities within Angkor’s settlement network (32–34) suggest that measurable change in land use intensity was highly asynchronous, akin to complex patterns of collapse and persistence across the Maya territories (35). This suggests historically and spatially contingent transformations within the Khmer state, and argues against discrete exogenous drivers. Our data contribute to the ongoing global debate surrounding the collapse of premodern states in relation to external stressors such as climatic variability (36–38) and emphasize the complexity of social transformation and adaptation.

While the breakdown of Angkor’s hydraulic network, most likely associated with climate variability in the mid-14th and early 15th centuries, represents the end of Angkor as a viable settlement, our data indicate that it was presaged by a protracted demographic decline. This raises the likelihood that the urban elite did not leave Angkor because the infrastructure failed, as has been suggested, but that the infrastructure failed (or was not maintained and repaired) because the urban elites had already left. The absence of Angkor’s ruling elite by the end of the 14th century casts a different light over the Ayutthayan occupation of the city from 1431 CE, and over Cambodian narratives that emphasize loss at the hands of interventionist neighboring states.

## Materials and Methods

**Fig. 4.**
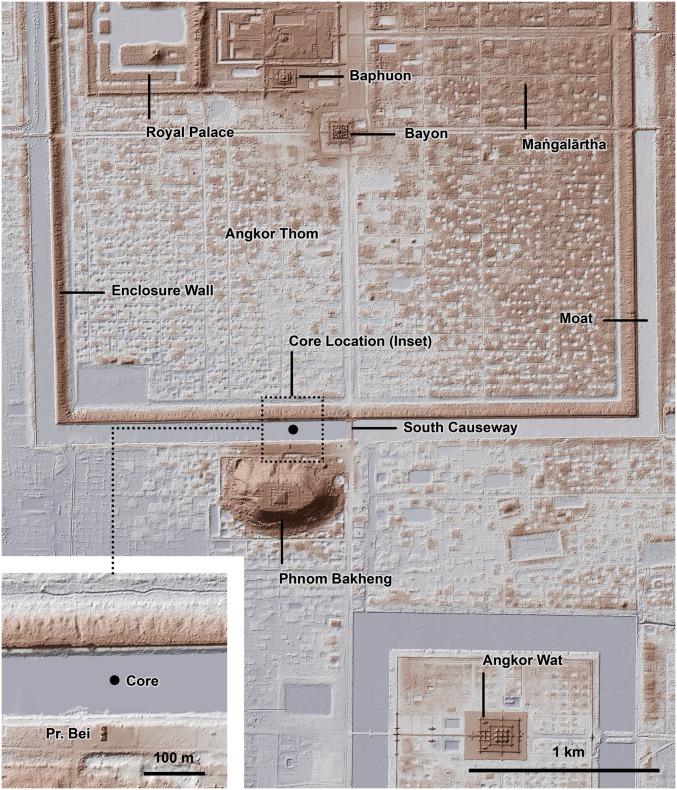
Lidar-based topographic map of central Angkor, showing the location of the core site in relation to features described in the text and the topographic evidence for intensive occupation both within and surrounding the moat and enclosure wall of Angkor Thom.

A core site was established ∼390 m west of the southern axial causeway of Angkor Thom, immediately north of the 10th-century brick temple Prasat Bei (Fig. 4), and ∼45 m north of the southern embankment of the moat (13°25′36.95″N, 103°51′24.24″E). A description of the area and its history is provided in *SI Appendix*. This sampling location places the cores south of the smaller 9th- to 11th-century moat identified by Gaucher (27, 28), but within the moat following its final renovation by Jayavarman VII in the 12th century. A coring platform was used to deploy a rope-operated percussion corer (39). Core samples were retained in 60-mm diameter PVC liners. It was necessary to remove the upper ∼20 cm of matted herbaceous vegetation to permit penetration of the core barrel. Four cores were taken from the site (AT/01/04/A, AT/01/04/B, AT/01/06/A, and AT/01/06/B), from adjacent locations (∼1 m apart along an E−W axis).

Volume magnetic susceptibility (κ) was measured on all cores in the field using a Bartington MS2 Meter with a 72-mm diameter MS2C core-scanning loop sensor to determine whether correlation between cores could be made and, therefore, whether a representative sample had been obtained (40). Correlation between replicate cores was established using a sequence slotting technique (41) based on volume magnetic susceptibility data from each core. Calculations were performed with CPLSlot 2.4b (42). Core liners were split longitudinally in the laboratory. All cores were described (43) and color recorded (44). The core was subsampled at contiguous 1-cm intervals for loss on ignition (ref. 45; LOI_550_ × 4 h following ref. 46), and palynological analysis (47). Pollen and spores were counted at 400 to 1,000 magnification using a Zeiss Axioskop microscope. Taxonomy was based primarily on reference material collected from vouchered plant specimens (National Herbarium of The Netherlands), and nomenclature followed ref. 48. Charcoal particles within the pollen preparations (therefore 200- to 7-μm fraction) were counted, and their absolute abundance was calculated (49). The absolute abundance of pollen grains and spores was calculated in the same manner.

Nine subsamples (×5.48 g wet weight) were taken from core AT/01/04/B for accelerator mass spectrometry radiocarbon dating, using pollen as the target fraction. Pollen was extracted from these samples following ref. 50, with the exception that the samples were initially sieved at 63 μm rather than 180 μm, and no other sieving was conducted. A radiocarbon standard (0.367 g dry weight of Hohenheim oak; FIRI code H; consensus value 2,232 ± 5 y B.P.; ref. 51) was pretreated with the sediment samples and dated so as to identify any laboratory-based contamination. Radiocarbon ages were calibrated using Calib 7.10 (IntCal13; ref. 52), and a chronological model was developed, using Bacon 2.2 (29).

Stratigraphically constrained cluster analysis was used to identify points of significant change in the data and was performed with an incremental sum-of-squares method (53) on the squared Euclidian distances of absolute pollen abundances using the Rioja package (54) in R 3.3.1 (55). Comparison of residual variance in the data with a “broken stick” model is used to determine the number of “significant” group boundaries within the pollen sequence (56). PCA was used to decompose absolute pollen and charcoal data into four components. All variables were normalized using division by their SD. All calculations were performed using PAST Software v. 3.13 (57).

## Supplementary Material

Supplementary File
