# Supplementary material for: Geoarchaeological evidence from Angkor, Cambodia, reveals a gradual decline rather than a catastrophic 15th-century collapse
Source: Proc Natl Acad Sci U S A. 2019 Feb 25;116(11):4871–6. doi: 10.1073/pnas.1821460116 (PMC6421400; doi:10.1073/pnas.1821460116)
Supplement: Supplementary File [file pnas.1821460116.sapp.pdf]

PNAS
www.pnas.org

Supplementary Information for

Geoarchaeological evidence from Angkor, Cambodia: a gradual decline in occupation rather than catastrophic 15th century collapse

Dan PENNY, Tegan HALL, Damian EVANS, Martin POLKINGHORNE

Corresponding Author: Dan Penny
Email: dan.penny@sydney.edu.au

This PDF file includes:

- Supplementary text
- Figs. S1 to S4
- Table S1
- References for SI reference citations

www.pnas.org/cgi/doi/10.1073/pnas.1821460116

Supplementary Information Text

Site Description

The area surrounding Phnom Bakheng, located between the 6th century city centred on Ak Yum to the west and the 8-9th century city of Hariharālaya to the south-east, has been a focus of settlement for millennia (Fig. 1). Groslier (*1*) reported, from extensive excavations he led in 1966, the occurrence of an Iron-Age site adjacent to early 10th century of the Common Era (CE) monument of Prasat Baksei Chamkrong. This was subsequently confirmed by a test-pit excavation in the same area (*2*). Phnom Bakheng became the focus for urban life at Angkor from the late 9th century, when king Yasovarman I established his new capital on and around the hill.

There are several phases in the development of Angkor Thom's moat, beginning in the 9th century with a relatively narrow and shallow moat closer to the enclosure wall, revealed in excavations on the eastern side of the south gate. The moat was likely widened and deepened to its final form in the late 12th century (*3*). The formalised defensive enclosure represented by Jayavarman VII's final renovation of the moat, then, represents the end of three centuries of continuous occupation and development (*4*). After the construction of Angkor Thom in the 12th century the area remained the very heart of Angkor, and the area around Angkor Thom's south gate was reported to be a thriving market by the mid-14th century Chinese trader Wang Dayuan (Wang Ta-yüan) (*5*; p. 105, 107-108), although the veracity of that account and its translation awaits serious critical analysis. After the 15th century the temple of Phnom Bakheng underwent immense restoration to produce a monumental seated Buddha (*6*) and also received pilgrims from Ayutthaya (*7, 8*).

High-resolution topographic mapping of central Angkor (Evans, 2016; Evans and Fletcher 2015; Evans et al. 2013) has revealed high-density occupation within and surrounding the enclosure of Angkor Thom (Figure 2), presumably contemporary with and post-dating the establishment of the walled city in the mid 12th century. This central area would have had a residential population of non-rice-producing people numbering in the hundreds of thousands (*9, 10, 11*).

Core description

Core AT/01/04/B is 70 cm in length and is composed of four stratigraphic units. The basal Unit 1 (70-64 cm depth) is a light grey (10YR 7/1) sandy clay with many yellowish brown (10YR 5/6) mottles in the form of clearly defined iron masses throughout the matrix. Fine to coarse, angular to sub-angular gravels are present. This is very-abruptly overlain by Unit 2 (64-44 cm depth), a

medium bedded dark-grey (2.5Y 4/1) sandy-clay. Unit 3 (44-14 cm depth) is a very dark grey (10YR 3/1) medium bedded, highly organic clay bed with a very abrupt lower boundary and very few distinct clay masses (10YR 4/1 and 10YR 4/2) of spherical shape and medium to coarse size (8-32 mm). Unit 4 is a black (10YR 2/1) fibrous peat (14-0 cm depth).

Description of stratigraphic analyses

Loss on ignition data for AT/01/04/B indicate consistently low organic carbon values in stratigraphic units 1 and 2 (average of 3.3 $\pm$ 0.83 % organic carbon by dry weight), progressively increasing through Unit 3 as depth decreases. Average organic carbon values are 54% by dry weight in Unit 4, reaching a maximum for the entire record of 80% organic carbon at 2 cm depth.

Mineral bulk density (kg/m^3; Figure 3) decreases as depth decreases, mirroring the increase in organic carbon in the sediment over time. Mineral accumulation rates are relatively low and stable in Units 1 and 2 (an average of 112.4 $\pm$ 23 kg/m^2/yr^{-1}), before increasing dramatically to an average of 458.8 $\pm$ 194.7 kg/m^2/yr^{-1} in Unit 3. Mineral influx declined gradually from 29cm depth, but fell precipitously from 18 cm depth, and average influx rates were only 75.9 $\pm$53.8 kg/m^2/yr^{-1} in Unit 4, well below the long-term average.

Description of palynology and charcoal result

Microfossils are apparent from the early 11th century C.E. (54 cm depth in the core), but increase in abundance dramatically after the moat is excavated in the late 12th century C.E. (43 cm depth in core, at the boundary between CONISS groups 1 and 2) reflecting better preservation associated with reducing conditions after the moat flooded. From the creation of the moat until the early decades of the 14th century the dryland vegetation was characterized by a changing admixture of taxa with no clear dominant. Pollen derived from the Dipterocarpaceae (*Dipterocarpus*, *Hopea/Shorea*), which dominate the dry- to mixed-deciduous forests in the area (*12*), are present in trace amounts throughout the record. We take this to reflect the extreme under-representation of these taxa in pollen assemblages related to their pollination strategies (*13*), rather than a reflection of its absence from the local vegetation. The rest of the assemblage is characterized by taxa such as Cannabaceae (Celtidoidae *sensu 14*; possibly *Celtis* but most probably *Gironniera* based on their respective habitat preferences), Fagaceae (particularly *Lithocarpus/Castanopsis*), and *Macaranga*. The cultivated palm *Borassus flabellifer* (*15*) is present, though highly variable, throughout the record. Grasses dominate the pollen assemblage derived from herbaceous and aquatic plants until the early decades of the 14th century (18 cm depth in core). Above 18 cm depth in the core changes

in dryland vegetation, such as marked increases in the abundance of Urticaceae/Moraceae P3 and Combretaceae/Melastomataceae are coincident with dramatic changes in the aquatic flora, including ferns. Increases in the abundance of littoral aquatic herbs such as Cyperacaee and Liliaceae, and floating macrophytes such as *Nympoides*, are particularly notable, as is the epiphytic swamp-forest fern *Stenochlaena* and the psilate and verrucate monolete spore groups. Grass pollen increases progressively in abundance through this period, reaching maximum values in the upper-most 3 cm of the record.

Microcharcoal particles vary dramatically over depth. Expressed as variance around the long-term mean (Figure 4), it is clear that charcoal particles are more abundant on average, and more highly variable on a sample-to-sample basis, from the excavation of the moat in the early 12th century (45 cm depth) until the first decades of the 14th century (17 cm depth). Above 17 cm depth variability in the concentration of charcoal particles falls markedly (standard deviation of samples below 17 cm depth is nearly twice that above 17 cm depth – $1.35x10^6$ v $7.3x10^5$). Charcoal concentrations also fall below the long-term mean from this depth, with the exception of a single spike in charcoal concentrations at 8 cm depth (circa 1400 CE).

Table S1: Results of radiocarbon dating of core AT/01/04/B

Lab. Code	Depth (cm)	$\delta(^{13}C)$ per mil	Percent modern $\pm$ 1σ error	Radiocarbon age (^{14}C years BP $\pm$ 1 σ)	Calibrated age CE (1 σ 68.3% probability)	Calibrated age CE (2 σ 95.4% probability)	Median Probability (cal years CE)
OZI290	7-8	-25.0*	91.76 $\pm$ 0.53	690 $\pm$ 50	1268- 1308 [0.67]<br>1361- 1386 [0.33]	1228- 1231 [0.003]<br>1246- 1332 [0.63]<br>1337- 1398 [0.37]	1301
OZH171	14-15	-27.0	94.50 $\pm$ 0.37	455 $\pm$ 35	1424- 1453	1409- 1487 [0.99]<br>1604- 1607 [0.003]	1440
OZI291	15-16	-25.0*	90.21 $\pm$ 0.49	830 $\pm$ 45	1169- 1176 [0.07]<br>1181- 1255 [0.93]	1049- 1084 [0.07]<br>1124- 1136 [0.02]<br>1150- 1276 [0.92]	1206
OZH172	17-18	-24.9	91.75 $\pm$ 0.34	690 $\pm$ 30	1275- 1299 [0.81]<br>1370- 1379 [0.19]	1266- 1312 [0.73]<br>1358- 1387 [0.27]	1291
OZI292	25-26	-25.0*	91.23 $\pm$ 0.42	740 $\pm$ 40	1247- 1289	1215- 1301 [0.97]<br>1368- 1381 [0.03]	1267
OZI293	35-36	-25.0*	92.44 $\pm$ 0.38	630 +35	1295- 1319 [0.38]<br>1351- 1391 [0.62]	1287- 1399	1349
OZH173	42-43	-22.7	90.01 $\pm$ 0.37	845 $\pm$ 35	1162- 1224 [0.93]<br>1235- 1241 [0.07]	1051- 1082 [0.06]<br>1128- 1134 [0.01]<br>1151- 1264 [0.93]	1197
OZI294	45-46	-25.0*	88.80 $\pm$ 0.52	955 $\pm$ 50	1024- 1054 [0.29]<br>1078- 1153 [0.71]	993- 1189	1093
OZI295	61-62	-25.0*	78.66 $\pm$ 0.58	1930 $\pm$ 60	18- 14 BCE [0.01]<br>0- 133 CE [0.99]	BCE 48- cal CE 231	73
OZI296	FIRI ‘H’	-23.0*	75.95 $\pm$ 0.37	2210 $\pm$ 40	-	-	

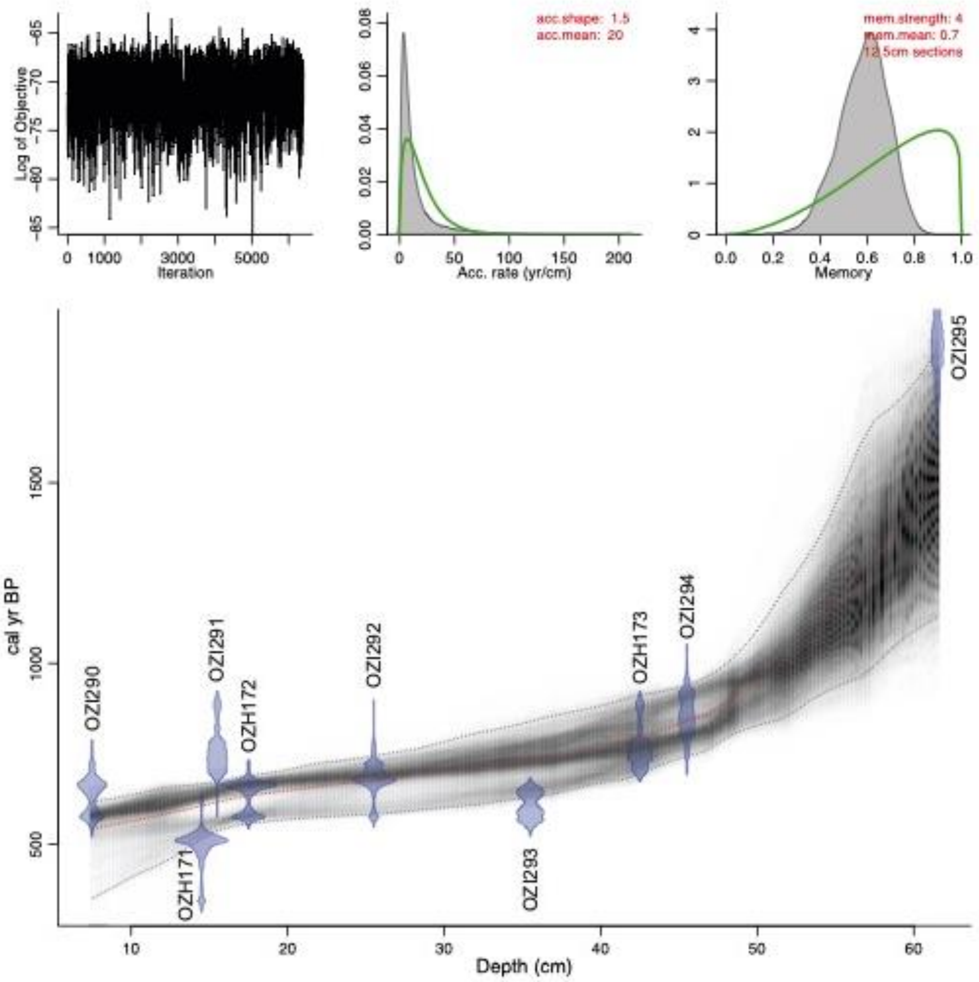

Fig. S1. Output of radiocarbon calibration and chronological modelling for core AT/01/04/B.

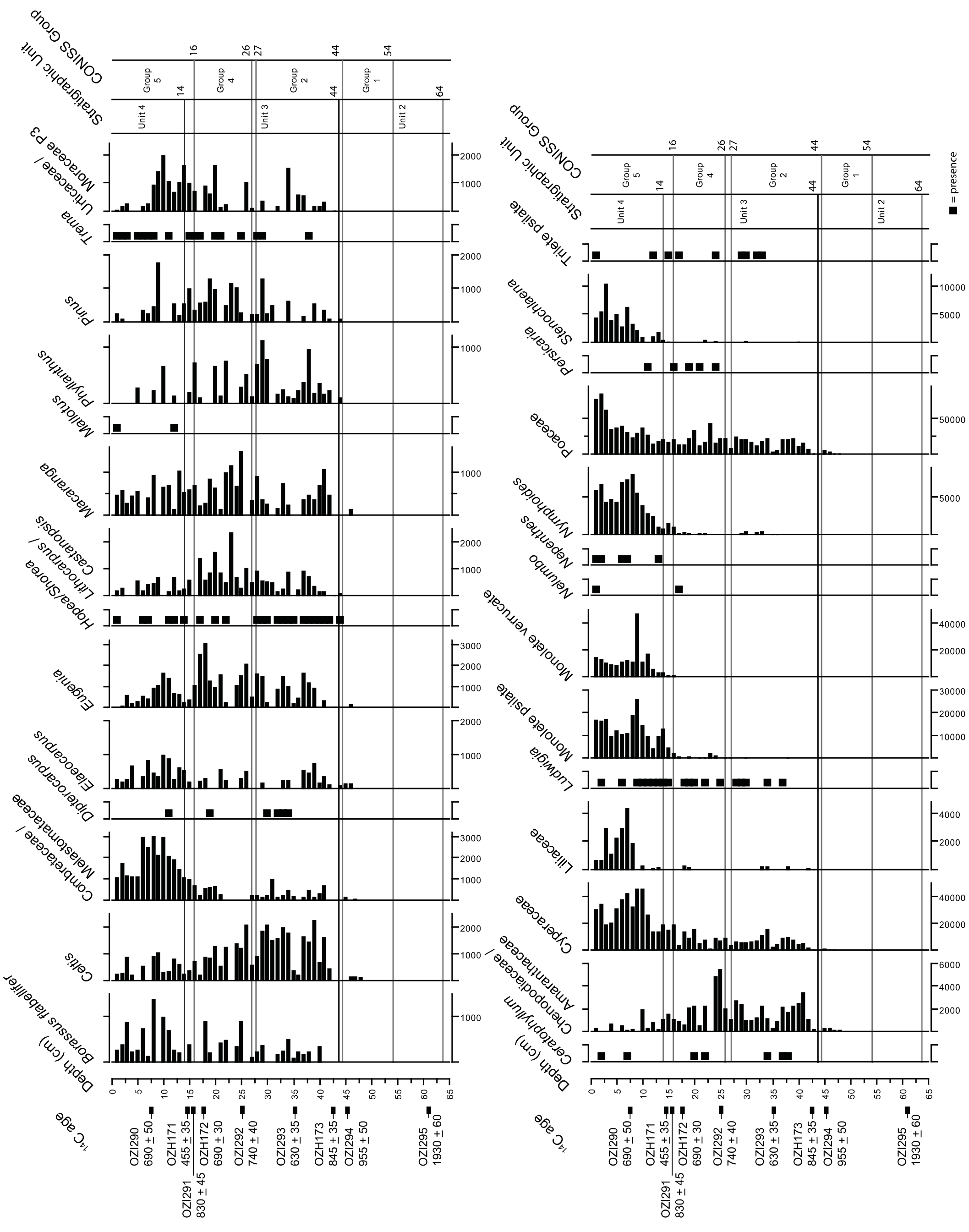

Fig. S2. Selected pollen and spore taxa, plotted against depth as absolute abundances for core AT/01/04/B. Dryland taxa (trees and woody shrubs) are shown in the upper (left) panel, and herbs, aquatics and ferns are shown in the lower (right) panel.

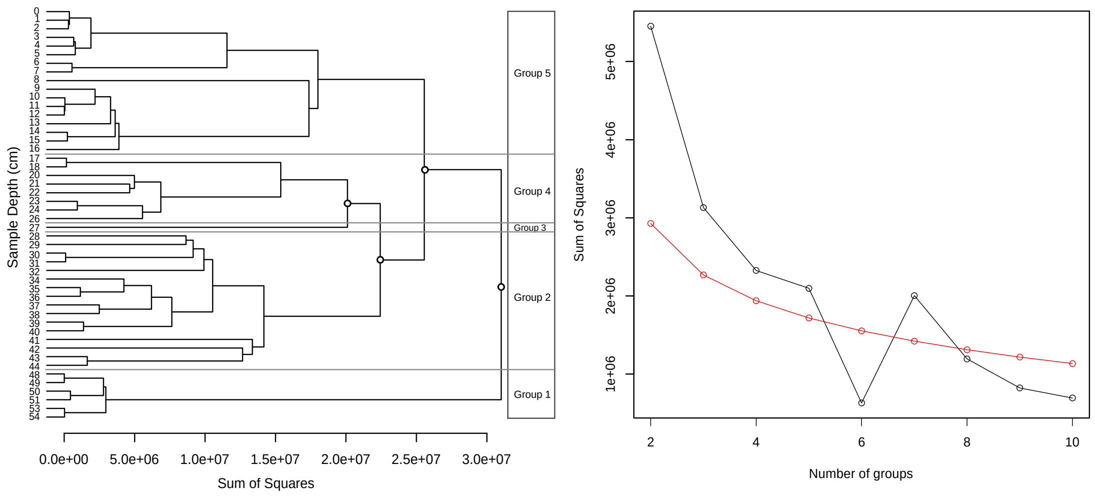

Fig. S3. Stratigraphically constrained cluster analysis of pollen data in absolute abundances. The first four divisions in the cluster are marked with open circles. Right panel shows comparison between residual dispersion of summed absolute pollen data with a broken stick model, indicating the number of sample groups that can be considered ‘significant’.

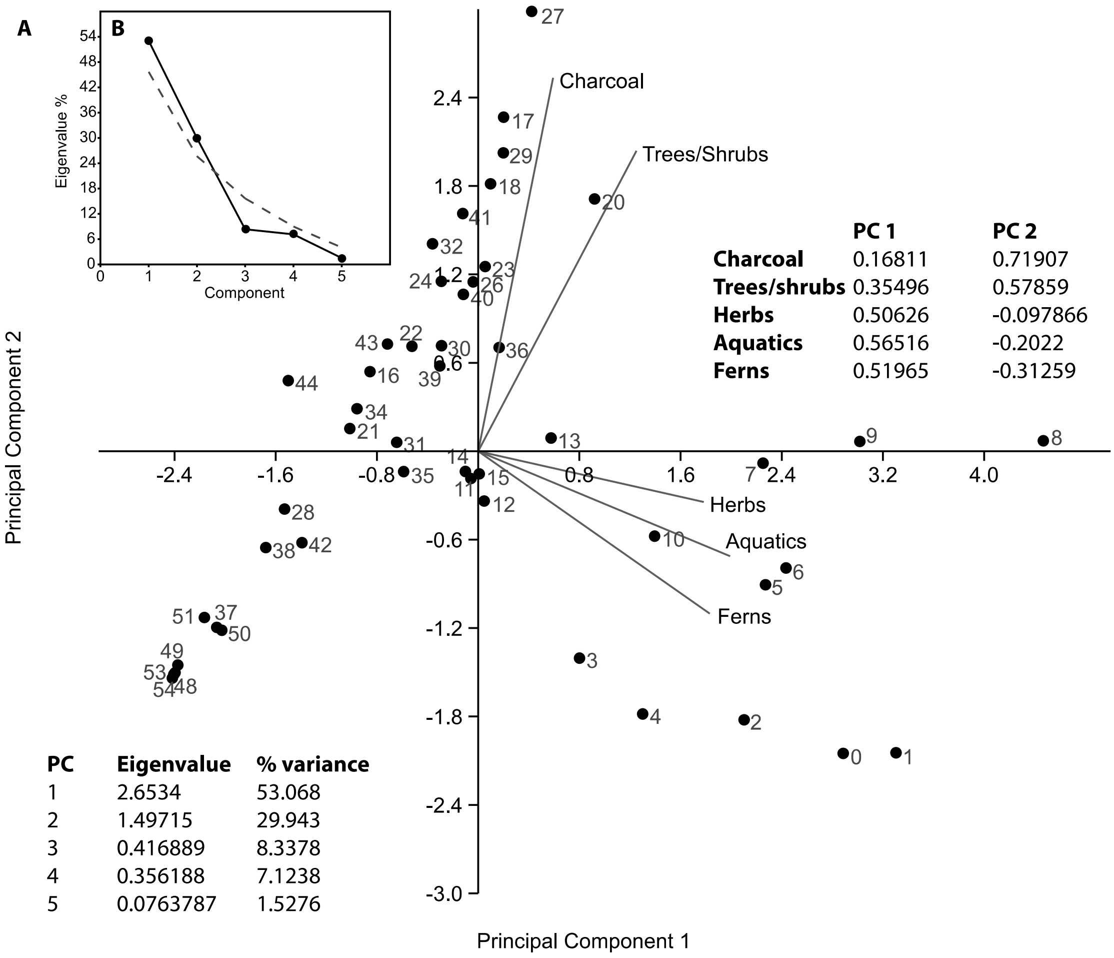

Fig S4: A) Result of Principal Component Analysis of pollen and charcoal data showing dispersion of samples relative to two principal component axes and, B) 'Broken Stick' model results that indicate only the first two component axes can be considered significant.

References

1. Groslier, B.P., 1979. La cité hydraulique angkorienne: exploitation ou surexploitation du sol?. *Bulletin de l'École française d'Extrême-Orient*, *66*, pp.161-202.
2. Higham, C.F.W, Thorsarat, R., 2001. A test excavation at Baksei Chamkrong, Angkor. *UDAYA* 2: 109-116.
3. Gaucher, J. 2017. L'Enceinte d'Angkor Thom: archéologie d'une forme, chronologie d'une ville. In Beschaouch, A., Verellen, F., Zinl, M. (eds.) *Deux décennies de coopération archéologique franco-cambodgienne a Angkor*. Académie des Inscriptions et Belles-Lettres, Paris : 27-42.
4. Gaucher, J. 2013 Considérations nouvelles sur la fondation d'Angkor Thom. In Pierre Baptiste and Thierry Zéphir (eds.), *Angkor, naissance d'un mythe : Louis Delaporte et le Cambodge*, Paris, Gallimard, p. 215-219.
5. Rockhill, W. W. (1915). Notes on the Relations and Trade of China with the Eastern Archipelago and the Coast of the Indian Ocean during the Fourteenth Century. Part II. *T'oung Pao*, *16*(1), 61-159.
6. Dumarçay, J. 1971. Phnom Bakheng. Étude Architecturale du Temple. *Mémoires Archéologiques VII*. École française d'Extrême-Orient: Paris.
7. Khin Sok. 1978. "V. Deux inscriptions tardives du Phnom Bàkhèn, K.465 et K.285", *Bulletin de l'École française d'Extrême-Orient* 60(1): 271-280.
8. Pou Saveros. 1989. Inscription du Phnom Bakheng (K.465), *Nouvelles inscriptions du Cambodge*, pp. 20-25, École française d'Extrême-Orient, Paris.
9. Evans, D., 2016. Airborne laser scanning as a method for exploring long-term socio-ecological dynamics in Cambodia. *Journal of Archaeological Science*, *74*, pp.164-175.
10. Evans, D. and Fletcher, R., 2015. The landscape of Angkor Wat redefined. *Antiquity*, *89*(348), pp.1402-1419.
11. Evans, D.H., Fletcher, R.J., Pottier, C., Chevance, J.B., Soutif, D., Tan, B.S., Im, S., Ea, D., Tin, T., Kim, S. and Cromarty, C., 2013. Uncovering archaeological landscapes at Angkor using lidar. *Proceedings of the National Academy of Sciences*, *110*(31), pp.12595-12600
12. Ashwell, D. (1993) *Zoning and Environmental Management Plan for Angkor: background report on the vegetation ecology of Angkor and environs.* IUCN/ UNESCO.
13. Bera, S.K., 1990. Palynology of *Shorea robusta* (Dipterocarpaceae) in relation to pollen production and dispersal. *Grana*, *29*(3): 251-255.
14. Zavada M. S. (1983) Pollen morphology of Ulmaceae. *Grana* 22: 23-30.
15. Penny, D., Pottier, C., Fletcher, R.J., Barbetti, M.F., Fink, D., Hua, Q., 2006. Vegetation and land-use at Angkor, Cambodia: a dated pollen sequence from the Bakong temple moat. *Antiquity* 80 (309): 599-614
